# Supplementary material for: A systematic review and meta-analysis on the efficacy of glibenclamide in animal models of intracerebral hemorrhage
Source: PLoS One. 2023 Sep 27;18(9):e0292033. doi: 10.1371/journal.pone.0292033 (PMC10529582; doi:10.1371/journal.pone.0292033)
Supplement: Attachment — Submitted filename: Response to Reviewers.docx [file pone.0292033.s003.docx]

**Academic Editor:**

1. Please elaborate more in detail the exclusion criteria of each excluded paper.
   - We have elaborated and added reasons and citations for each excluded paper in our Search Results section on page 14, lines 324-328. Further, we have added in justification for the exclusion of pre-ICH dosing regimens on page 9, lines 212-215.
2. On Introduction, page 5, lines 89-94 please elaborate more on the possible mechanisms that may explain the controversial findings in reference to reduction of brain edema and its effects on functional outcome and mortality of ICH.
   - We have added this information in on pages 5-6, lines 112-124.
3. Page 11, lines 110-114. Even if many experimental therapies seem to function in animal models of acute stroke, the translational gap should not be forgotten. The therapy options for ischemic stroke in the clinical setting are restricted to thrombolysis and trombectomy in selected patient cohorts.  Please correct and reformulate the text.
   - We thank the editor for noting this misleading wording. We have fixed our wording in the original sentence on page 7, lines 146-147, and have added in a separate line clarifying that thrombectomy and thrombolysis remain the only clinically approved therapies for ischemic stroke on page 6, lines 138-140.
4. Please also reformulate the ambiguous and misleading sentence on line 113 "Notably, unlike ischemic stroke, serum extrusion is a major cause of edema in ICH, which GLC should not affect".
   - We have clarified our wording in this sentence on page 7, lines 150-152.

**Reviewer #1**

The author wrote about the effect of Glibenclamide in animal models of intracerebral hemorrhage. The paper exhibits a well-written and comprehensive investigation into the intricate relationship between Glibenclamide and ICH in animal models. The authors have provided an extensive discussion, which significantly contributes to our understanding of this important research area.

Despite the shortage of analyzed studies, the paper demonstrates a commendable level of scientific rigor in its experimental design and data analysis. The methodology employed in this study is robust, and the systematic review procedures are meticulously described, facilitating reproducibility. The authors' discussion showcases a comprehensive understanding of the topic, as they skillfully interpret the experimental results and effectively relate them to existing knowledge in the field.

However, there are several aspects that need to be explained more. We encourage the authors to address the following aspects to strengthen the impact of their study and ensure its relevance to the broader scientific community:

1. In the excluded full papers list, the author mentioned about “four papers were excluded for inappropriate patient populations, and one for using an excluded dosing regimen” Can the author explain more about this reason?
   - We have elaborated and added reasons and citations for each excluded paper in our Search Results section on page 14, lines 324-328. Further, we have added in justification for the exclusion of pre-ICH dosing regimens on page 9, lines 212-215.
2. The author needs to add an introduction about the importance of defining a new treatment to reduce the edema since the use of diuretics is common in clinical practice. The use of glibenclamide in reducing edema in human patients with ICH and its comparison with common diuretics that are used to treat edema are interesting aspects to be compared.
   - We have added in a paragraph elaborating on the treatment of edema in ICH and included the most common clinical management methods on page 7, from lines 153-169. As HTS and mannitol were not investigated in our systematic review, the section was kept brief, with references cited to guide readers who may be interested in reading about these therapies in more detail.
3. It is mentioned in line 113 "Notably, unlike ischemic stroke, serum extrusion is a major cause of edema in ICH, which GLC should not affect". This line is mismatched with the reason why this systematic review needs to be done. How does the author explain or elaborate on this?
   - We thank the reviewer for noting our confusing wording. Highlighting serum extrusion as something that occurs in ICH but not in ischemic stroke was meant to reinforce the importance of original research on GLC specifically in the context of ICH, rather than the meta-analysis itself. Due to key differences between stroke subtypes, it remains important that ischemic stroke results not be used to justify the use of GLC in ICH patients. We have elaborated on this in the manuscript on page 7, lines 147-152.
